# Rapid GMP-Compliant Expansion of SARS-CoV-2–Specific T Cells From Convalescent Donors for Use as an Allogeneic Cell Therapy for COVID-19

**DOI:** 10.3389/fimmu.2020.598402

**Published:** 2021-01-08

**Authors:** Rachel S. Cooper, Alasdair R. Fraser, Linda Smith, Paul Burgoyne, Stuart N. Imlach, Lisa M. Jarvis, David M. Turner, Sharon Zahra, Marc L. Turner, John D. M. Campbell

**Affiliations:** ^1^ Tissues, Cells and Advanced Therapeutics, Scottish National Blood Transfusion Service, Edinburgh, United Kingdom; ^2^ National Microbiological Reference Unit, Scottish National Blood Transfusion Service, Edinburgh, United Kingdom; ^3^ Histocompatibility and Immunogenetics, Scottish National Blood Transfusion Service, Royal Infirmary of Edinburgh, Edinburgh, United Kingdom

**Keywords:** COVID-19, T cell, adoptive T cell immunotherapy, CD4, CD8, memory T cell

## Abstract

COVID-19 disease caused by the SARS-CoV-2 virus is characterized by dysregulation of effector T cells and accumulation of exhausted T cells. T cell responses to viruses can be corrected by adoptive cellular therapy using donor-derived virus-specific T cells. One approach is the establishment of banks of HLA-typed virus-specific T cells for rapid deployment to patients. Here we show that SARS-CoV-2–exposed blood donations contain CD4 and CD8 memory T cells which recognize SARS-CoV-2 spike, nucleocapsid and membrane antigens. Peptides of these antigens can be used to isolate virus-specific T cells in a GMP-compliant process. The isolated T cells can be rapidly expanded using GMP-compliant reagents for use as an allogeneic therapy. Memory and effector phenotypes are present in the selected virus-specific T cells, but our method rapidly expands the desirable central memory phenotype. A manufacturing yield ranging from 10^10^ to 10^11^ T cells can be obtained within 21 days culture. Thus, multiple therapeutic doses of virus-specific T cells can be rapidly generated from convalescent donors for potential treatment of COVID-19 patients.

## Introduction

Coronavirus disease 2019 (COVID-19), caused by the severe acute respiratory virus syndrome-coronavirus 2 (SARS-CoV-2) emerged in Wuhan, China in December 2019. In the majority of cases infection with SARS-CoV-2 is asymptomatic or leads to relatively mild self-limiting disease, but a proportion of patients progress to severe disease with about a 1% overall mortality rate (1, 2). Declared a pandemic by the WHO on March 11, 2020, the virus has spread rapidly to all parts of the world with >56 million infections and >1.35 million deaths reported by November 2020 (3).

Patients with progressive severe disease demonstrate a high neutrophil to lymphocyte ratio and a lymphopenia in the blood accompanied by a hyperinflammatory and prothrombotic diathesis leading to Acute Respiratory Distress Syndrome (ARDS) and multiorgan failure (4–6). Some success in treating severe disease has recently been reported with therapeutic agents such as remdesivir (7), dexamethasone (8), and nebulized interferon-beta (9).

A particular feature of progressive COVID-19 disease is rapid exhaustion of the memory T cell compartment—characterized by overall lymphopenia and accumulation of naïve/exhausted T cell memory phenotypes (10, 11). This undesirable phenotype is associated with a systemic hyperinflammatory response and poor outcomes (reviewed in (12)). Conversely, protection in self-limiting disease is associated with strong CD4 and CD8 T cell responses to the spike, membrane and nucleocapsid proteins of the virus and development of virus-specific antibodies (13–15). Convalescent plasma (CP) is currently being trialed in a number of countries as a potential therapeutic option, although the level and duration of protection afforded by the antibody response against re-infection remains unclear at present (16).

New therapies to support the immune response to SARS-CoV-2, preventing the collapse of the lymphocyte compartment and supporting protective immunity would have significant impact on outcome for hospitalized patients. Anti-viral T cells specific for viruses such as cytomegalovirus (CMV), adenovirus (ADV) and Epstein Barr Virus (EBV) have been successfully used as adoptive cellular therapies to combat such infections in patients with immune deficiency (17–22). Following selection of antigen-specific T cells from a blood donation from an individual who has been infected with the relevant virus, T cells may be administered to the patient without further manipulation. An alternative strategy is to expand virus-specific T cells *in vitro* using donations from HLA-typed donors. These T cells can be cryopreserved from multiple donors as an allogeneic “off the shelf” therapy and are typically used as a “best-HLA match” to the recipient. We and others have adopted this approach in the treatment of EBV or CMV-driven disease (21, 22) with evidence of *in vivo* efficacy, disease remission and low incidence of Graft versus Host Disease (GvHD) (21, 22). Despite a number of *in silico* studies (23), more data are required on HLA restriction of SARS-CoV-2 peptides in different populations to understand which HLA alleles and loci should be preferentially matched between donors and patients to optimise the efficacy of SARS-CoV-2 T cell therapy.

In this study we present clear evidence to show that donations from individuals who have been infected with SARS-CoV-2 with mild symptoms and have recovered retain normal T cell compartment profiles, with CD4 and CD8 memory and effector T cells specific for SARS-CoV-2 spike, nucleocapsid and membrane antigens. These virus-specific T cells (VSTs) can be isolated using Good Manufacturing Practice (GMP)-compatible selection technology and rapidly expanded *in vitro* using closed culture vessels and GMP-compliant reagents and medium. The mononuclear cell fraction of a single whole blood donation from a COVID-19 convalescent donor (CCD) can be used to generate up to 10^11^ T cells within 21 days with the desired central memory phenotype as a potential new therapy for SARS-CoV-2. This offers the potential for the manufacture of a bank of HLA-matched donor T cell products for use in clinical trial and future treatment of COVID-19 patients.

## Materials and Methods

### Study Design

The aim of this study was to characterize the SARS-CoV-2 peptide-specific T cell memory populations present in donations from CCD and to explore the feasibility of isolating and expanding these T cells to clinical scale. The expanded T cells could then form the basis of an HLA-typed allogeneic ‘off the shelf’ VST therapy for COVID-19. SNBTS is leading the Scottish COVID-19 convalescent plasma program, and COVID-19 Convalescent Donors (CCD) were also recruited from the local Scottish population to donate peripheral blood buffy coats for this study. CCD were eligible to donate if they had a confirmed positive SARS-CoV-2 PCR test and were a minimum of 28 days after resolution of infection symptoms, as well as fulfilling the current criteria for whole blood donation. Uninfected Donors (UD - adults confirmed as having no evidence of COVID-19 symptoms at time of donation) were used to compare initial phenotyping and SARS-CoV-2 antigen T cell responses with CCD. Buffy coats from CCD (n = 15) and UD (n = 17) were obtained under SNBTS Sample Governances 20~02 and 19~11 respectively. All donations were fully consented for research use.

### SARS-CoV-2 Antibody Detection

The Euroimmun anti-SARS-CoV-2 assay (Euroimmun US, NJ, USA) clinical diagnostic indirect ELISA was used to detect antibodies to SARS-CoV-2 spike protein from donor serum according to the manufacturer’s instructions. The results were expressed as a ratio against a calibrator control, where values of <0.8 were considered negative and >1.1 were considered positive.

### Buffy Coat Peripheral Blood Mononuclear Cell (PBMC) Isolation

Buffy coats were diluted [1:3] with PBS and added to Leukosep tubes containing Ficoll-Paque (GE Healthcare). Tubes were centrifuged at 450*g* for 40 min and the resulting buffy layer extracted. Isolated PBMCs were then washed in PBS and counted on MACSQuant10 Analyzer (Miltenyi Biotec).

### Scale-Up to Representative Manufacturing Process

For full-scale clinical manufacturing the starting material was taken from leukapheresis collections with no requirement for Ficoll preparation. Two demonstration products were generated using commercially-acquired leukapheresis material (5L Optia process, supplied by Key Biologics Ltd/Cellero, TN, USA) from CCD. Alternatively, automated devices such as the Sepax device (GE Healthcare) to Ficoll buffy coat to make a mononuclear fraction could be used for manufacturing if leukapheresis is not available (24).

### HLA Typing

Following extraction of DNA from PBMC samples, HLA genotyping of donors was undertaken for HLA-A, B and DRB1 loci using Lifecodes HLA eRES SSO Typing kits (Immucor Inc, USA).

### Immunophenotyping

Freshly isolated PBMC and T cells from VST cultures (see below) were analyzed for surface immunophenotype. For this, 2 × 10^6^ cell samples were taken and washed with PBS buffer supplemented with EDTA and human serum albumin (PEA buffer). Cell pellets were re-suspended in 100 μl PEA and incubated with 5 μl Fc Receptor blocking reagent to prevent non-specific antibody binding. Antibody surface marker multi-color panels detailed in **Figure S1** were then added for 20 min at 4°C. Samples were then washed and re-suspended in PEA, with dead cell dye DRAQ7 (eBioscience) added prior to acquisition on a MACSQuant10 Analyzer (Miltenyi Biotech) recording a minimum of 100,000 events.

### SARS-CoV-2 VST Detection Within PBMC Population

SARS-CoV-2 Peptivator peptide pools (Miltenyi Biotech) containing 15-mer sequences with 11 amino acids overlap for the immunodominant section of the spike protein, and the full sequence for nucleocapsid protein and membrane protein (**Table S1**) were reconstituted in DMSO/water according to manufacturers’ guidelines.

PBMC were plated in TexMACS medium (Miltenyi Biotech) in a 24-well plate at 5 × 10^6^ cells/ml per well with treatments: negative control, PMA/ionomycin positive control, individual spike, nucleocapsid, and membrane peptide pools, and combined pools (spike + nucleocapsid + membrane [SNM]). SARS-CoV-2 peptide pools were used at (0.3 nmol/ml), and cell activation cocktail (BioLegend) added to the positive control well at (1×). The negative control well contained DMSO/water at the same volume as the peptide wells. Cells were stimulated for a total of 5 h at 37°C, 5% CO2; with Brefeldin A (BioLegend) added at (5 μg/ml) for the final 3 h (Brefeldin A was only used in this analysis, not in any cell selection experiments).

### Intracellular Labeling

Plates were harvested into FACS tubes and washed with PEA. Samples were treated with Fc Receptor blocking reagent as above, and surface marker antibodies for multi-color panels (see **Figure S2** for details) were then added for 20 min at 4°C. Cells were then washed and stained with fixable viability dye (FVD) eFluor780 (eBioscience) for 30 min at 4°C. Cells were subsequently fixed and permeabilized using Cytofix/perm kit (BD Biosciences) for 20 min at 4°C, then washed and labeled with antibodies for intracellular cytokines (detailed in **Figure S2**) for 20 min at 4°C. Cells were washed and analyzed with a MACSQuant10 Analyzer recording a minimum of 150,000 events.

### SARS-CoV-2 VST Isolation

SARS-CoV-2 VSTs were isolated from PBMC whole population using a cytokine capture system (CCS) assay. Briefly, PBMC were plated at 5 × 10^6^ cells/ml/cm^2^ in standard Corning multi-well plates and incubated overnight at 37°C, 5% CO_2_. The following morning, plates were stimulated with pooled SARS-CoV-2 peptide pools (spike + nucleocapsid + membrane) each at (0.3 nmol/ml) for 6 h at 37°C, 5% CO_2_. The virus-specific IFN-γ secreting cells were then isolated using the IFN-γ CCS assay by either manual column or CliniMACS Prodigy isolation as described in (17). Following isolation, each fraction was counted and phenotyped using the lymphocyte panel as described above and illustrated in **Figure S1**.

### SARS-CoV-2 VST Culture Optimization

Non-target cells from the IFN-γ CCS assay were irradiated at 40 Gy and used as feeders for the IFN-γ+ target cells. Cultures were initially seeded at either 1 × 10^7^ total cells per cm^2^ (200–400 non targets: 1 target), or 3 × 10^6^ total cells per cm^2^ (100 non targets: 1 target) in G-Rex culture vessels (Wilson Wolf). Cells were cultured in GMP-grade TexMACS medium, and supplemented to determine culture optima using (200 U/ml) IL-2 (GE Healthcare), (155 U/ml), IL-7 (Miltenyi Biotech), (2%) human AB Serum (SNBTS) or (2%) nLiven (Sexton Biotechnologies). As nLiven is not fully European GMP-compliant it was replaced with pathogen-inactivated, xeno-protein-free GMP-compliant T-Liven (Sexton Biotech) for the scale up demonstrator VST products from leukapheresis. Cells were cultured for up to 28 days with feeds every 3 to 4 days and cultures split as necessary to maintain a density of 0.5 to 3 × 10^6^ T cells/cm^2^. At day 14, VST cultures from six donors were split to test feeder re-stimulation, where thawed irradiated non-target cells were added to cultured VSTs at (10 non targets: 1 VST) alongside a control culture with no feeder re-stimulation. Samples were taken every 3 to 4 days for immunophenotyping with the lymphocyte panel described above.

### Representative Scale Manufacture of VST

For scale manufacture, the 14-day procedure was chosen to maximize T cell yield without the requirement for a second feeder cell co-culture step. Leukapheresis material was obtained from SARS-CoV-2 convalescent USA-based donors (KBL/Cellero) and 2 × 10^9^ MNC taken and processed for VST isolation using the Miltenyi Prodigy cell processor and the CCS isolation program. Combined peptide pools (6 ml starting volume) were prepared by passing through a 0.22-μm filter into a 20-ml transfer bag, which was attached to the prodigy tubing set TS500 *via* a sterile welder. The positive target fraction was assessed and the negative fraction irradiated as before, then the cells combined and cultured in G-Rex CS100M flasks at 3 × 10^6^ cells/cm^2^. Cells were counted and split at days 7 and 11, and flasks harvested at day 14 with closed processing used throughout (Gatherex, Wilson Wolf Ltd). The cells were then assessed for cell numbers, surface phenotype and functional response as before. The final product was frozen in 33% plasmalyte/67% CryoStor10 (CS10, Stem Cell Technologies).

### Generation of Monocyte-Derived Dendritic Cells (DC)

Monocyte-derived DC were generated from isolated PMBC CD14+ monocytes. Briefly, monocytes were isolated from purified PMBC using anti-CD14 microbeads (Miltenyi Biotech) as per manufacturer’s instructions. Cells were cultured at 37°C, 5% CO_2_ for 6 days in RPMI (Life Technologies) supplemented with (5%) AB serum, (2 mM) Glutamax (Sigma-Aldrich), (20 ng/ml) GM-CSF, and (15 ng/ml) IL-4 (both Miltenyi Biotech). Media was replaced on days 2 and 4 of culture. After 6 days, cells were harvested using (1×) TrypLE (Life Technologies) and frozen in CS 10 (Stem Cell Technologies) until required for VST stimulation.

### SARS-CoV-2 VST Stimulation Assay

Expanded T cells form the 5 complete optimized expansion runs starting with PBMC, and the 2 expansion processes using Leukapheresis material were taken at day 14 to test in a stimulation assay with peptide-loaded DC. Briefly, frozen autologous immature DC were thawed, plated with RPMI medium and stimulated with individual SARS-CoV-2 peptide pools and combined pools at (0.3 nmol/ml) for 6 h at 37°C, 5% CO_2_. Where possible, VSTs were tested for specificity to SARS-CoV-2 by testing against other virus-specific peptides (Adenovirus Hexon peptide, Epstein-Barr Virus consensus peptide pool, and GAD65 peptide, all Miltenyi Biotech). Unloaded DC were included as a negative control. DC were then washed and re-plated in RPMI supplemented with poly I:C (20 μg/ml) and PGE_2_ (1 μg/ml) at 2.5 × 10^5^ cells/cm^2^ overnight to drive DC maturation. The next morning DCs and VSTs were co-cultured at 2.5 × 10^6^/cm^2^ (10 VST: 1 DC). Control wells were included to measure baseline stimulation of VSTs co-cultured with unloaded DCs. Plates were then stimulated for 5 h and labeled for the cytokine and activation panels as described above.

### Flow Cytometry Data Analysis

Analysis of all flow cytometry data was performed using either MACSQuantify (Miltenyi Biotec) for cell counts, or FlowJo version 7 (TreeStar Inc) for wider phenotyping analysis. All analyses were subject to a basic initial gating strategy in which debris was first excluded on the basis of forward and scatter properties, and sequentially gated for singlets using FSC Area versus Height, and finally sequentially gated on live cells (DRAQ7 or FVD negative cells). Populations and acquisition of activation markers/cytokines were then quantified using percentages corrected to negative controls (see **Figures S1** and **S2** for full gating strategies).

### Statistical Analysis

Statistical analysis was performed using GraphPad Prism version 8.4.2 (GraphPad Software). Comparisons of population frequencies between healthy donors and COVID-19 convalescent donors were performed using unpaired two-tailed Student’s t tests with Holm-Sidak correction for multiple comparisons. Tests comparing population frequencies intra-donor between time-points (i.e. day 0 versus day 14, and day 14 versus day 21) for lymphocyte phenotype markers or comparing acquisition of activation markers within the CD8 versus CD4 population were paired two-tailed Student’s T t-tests, corrected for multiple comparisons using the Holm-Sidak method where relevant. Response comparisons between the three individual SARS-CoV-2 peptides (spike, nucleocapsid and membrane) were tested using repeated-measures one-way ANOVA with the Geisser-Greenhouse correction to assume equal variability of differences within VST cultures. Correlation between intra-donor frequency of populations compared to other baseline characteristics was done by computing linear correlation coefficients using Pearson’s correction with confidence intervals of 95%. Unless otherwise stated, data are represented as mean values ± SEM.

## Results

### Donor Characteristics and Leukocyte Phenotype

Buffy coats from CCD (n = 15, see **Table 1**) were collected from local Scottish donors between 34 and 56 days after resolution of symptoms (diagnosis and resolution of infection were confirmed by SARS-CoV-2 PCR). Donors were 23 to 58 years old (median, 49 years) and evenly split by gender (7 female, 8 male). In all cases, donors exhibited mild symptoms of COVID-19 infection and did not require hospital treatment. The HLA-A, B and DRB1 typing results are also shown in **Table 1** for 12/15 donors that gave specific consent for HLA typing to be carried out.

**Table 1:** Baseline characteristics of COVID-19 convalescent donors and immune response at donation.

Donor Code	Blood Group	HLA Type						Days from symptoms onset to resolution	Days from symptoms resolution to donation	Antibody level	% SARS-CoV-2 VST
		Ax	Ay	Bx	By	DRB1x	DRB1y				
C19BC1	O pos	31:01	32:01	35:01	51:01	01:01	13:01	8	38	10.5	0.22
C19BC2	O pos	01:01	68:01	51:01	57:01	07:01	14:01	10	36	5.9	0.22
C19BC3	A pos	*						–	34	9.3	0.13
C19BC4	O pos	03:01	26:01	07:02	38:01	15:01	16:01	13	45	3.1	0.12
C19BC5	O pos	03:01	32:01	07:02	27:03/05	08:01	13:01	14	52	3.9	0.03
C19BC6	A neg	01:01	01:01	08:01	08:01	03:01	07:01	14	55	2.3	0.05
C19BC7	O pos	*						14	56	6.2	0.07
C19BC8	B pos	02:01	11:01	07:02	56:01	01:01	08:01	10	46	9.5	0.10
C19BC9	O neg	02:01	11:01	18:01	44:02	01:01	15:01	18	37	7.4	0.16
C19BC10	O pos	**						–	–	11	0.05
C19BC11	B neg	01:01	26:01	08:01	44:02	03:01	04:01	7	49	11	0.26
C19BC12	A pos	02:01	24:02	35:01	44:02	04:01	04:04	7	53	9.42	0.13
C19BC13	O pos	*						–	–	5	0.08
C19BC14	O pos	02:01	02:01	NT	NT	04:04	15:01	–	–	4.51	0.07
C19BC15	O neg	03:01	29:02	44:03	57:01	07:01	15:01	13	53	3.48	0.03

Antibody level refers to Euroimmun assay values (>1.1 = positive). Percentage SARS-CoV-2 VST refers to percentage CD3+/IFN-γ+ cells responding to combined SARS-CoV-2 pooled peptides (see **Figure 2** for data analysis).

*Not consented for HLA typing.

**No sample taken for HLA typing.

NT, not tested as insufficient DNA to type locus.

**Figure 1:**
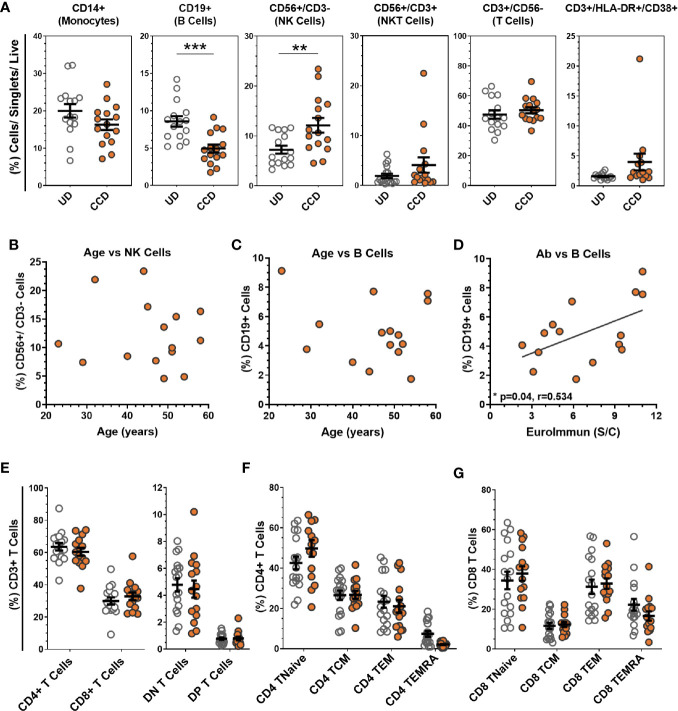
Analysis of COVID-19 convalescent donor buffy coat-derived PBMCs. **(A)** Buffy coat-derived PBMCs from COVID-19 convalescent donors (CCD, n = 15, orange circles) and healthy uninfected donors (HD, n = 17, clear circles) were assessed for leukocyte lineage by flow cytometry (see **Figure S1A** for gating strategy). No correlation was seen between age of COVID-19 convalescent donors with **(B)** NK cells or **(C)** B cells, but significant correlation between **(D)** SARS-CoV-2 serum antibody content and the percentage of B cells between donors (p = 0.04, r = 0.534, Pearson correlation coefficient). **(E)** Analysis of the T cell compartment (see **Figure S1B** for gating strategy) shows comparable mean levels of T cell subtypes, as well as **(F)** CD4+ and **(G)** CD8+ memory populations between HD and CCD. Data is represented as mean ± SEM. Significance determined by unpaired t-test with Holm-Sidak correction for multiple comparisons. DN double negative (CD4−/CD8−), DP double positive (CD4+/CD8+), TNaive (CD62L+/CD45RA+/CD45RO−), TCM central memory (CD62L+/CD45RA−/CD45RO+), TEM effector memory (CD62L−/CD45RA−/CD45RO+), TEMRA terminal effector memory CD45RA revertant (CD62L−/CD45RA+/CD45RO−). **p ≤ 0.01 and ***p ≤ 0.001.

Immunophenotyping of buffy coat-isolated peripheral blood mononuclear cells (PBMC) from CCD compared to uninfected donors (UD, n = 17) is shown in **Figure 1**. PBMC were sequentially gated as per **Figure S1**. The mean percentages of T cells (CD3+/CD56-), NKT cells (CD56+/CD3+) and monocytes (CD14+) were comparable between UD and CCD (**Figure 1A**). The mean percentage of T cells with an activated phenotype (HLA-DR+/CD38+), reported as elevated in other studies with moderate to severe disease, were not found to be significantly different between UD and CCD in this study. NK cell levels were significantly elevated (p = 0.0073) in CCD compared to UD and the mean percentage of B cells in CCD was significantly lower than UD (p = 0.0003). In this study, age did not correlate with NK cell or B cell levels in CCD (**Figures 1B, C**), though a significant correlation (Pearson correlation p = 0.04, r = 0.524) was identified between percentage of B cells and SARS-CoV-2 antibody content (**Figure 1D**). Within the T cell compartment the percentage of CD4 and CD8 T cells, as well as CD3+/CD4-/CD8- (double negative) and CD3+/CD4+/CD8+ (double positive) remained unchanged between CCD and UD (**Figure 1E**). In addition, analysis of co-expression of T cell memory markers CD62L, CD45RO, and CD45RA reveals no difference in CD4 and CD8 memory subpopulations between UD and CCD for either CD4 T cells (**Figure 1F**) or CD8 T cells (**Figure 1G**).

### CCD T Cell Responses to Spike, Nucleocapsid, and Membrane SARS-CoV-2 Peptides

**Figure 2:**
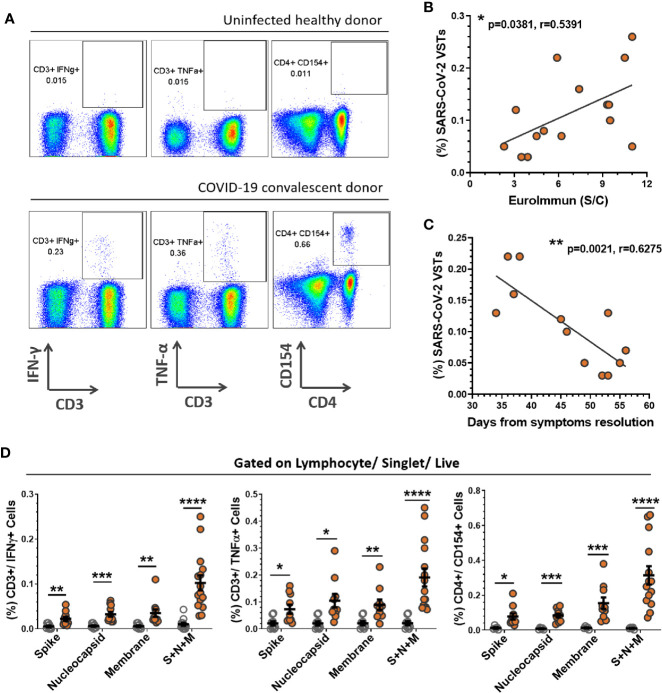
PBMC responses to SARS-CoV-2 peptides. PBMCs derived from buffy coats were incubated with SARS-CoV-2 peptides (Spike + Nucleocapsid + Membrane) for 5 h and corrected against a no antigen control well for positive expression of cytokines and activation markers. **(A)** Representation of flow cytometric analysis from a healthy uninfected donor (HD) and COVID-19 convalescent donor (CCD), note all flow analyses were gated on lymphocytes/single cells/live cells and subsequently quantified for percentage CD3+/IFN-γ+ cells, CD3+/TNF-α+ cells and CD4+/CD154+ cells (S2 for gating strategy). The percentage of SARS-CoV-2 VSTs in the CCD PBMC population (i.e. CD3+/IFN-γ+ cells reactive to pooled S+N+M peptides corrected to no antigen control) significantly correlated with **(B)** antibody titer at donation (p = 0.0381, r = 0.5391) and **(C)** days from resolution of symptoms to donation (p = 0.0021, r = 0.6275). Calculation was performed using Pearson correlation coefficient. **(D)** Mean percentages of CD3+/IFN-γ+ cells, CD3+/TNF-α+ cells and CD4+/CD154+ cells for individual and pooled peptides corrected to no antigen control were compared between HD (n = 12, clear circles) and CCD (n = 15, orange circles). Data is represented as mean ± SEM. Statistical significance was determined using unpaired t-tests corrected for multiple comparisons using the Holm-Sidak method where *p ≤ 0.05, **p ≤ 0.01, ***p ≤ 0.001 and ****p ≤ 0.0001.

PBMC were stimulated with SARS-CoV-2 peptide pools for spike protein, nucleocapsid protein and membrane glycoprotein or combined pools of all three and subsequently labeled for T cell surface markers (CD3, CD4, CD8) and intracellular cytokines (IFN-γ, TNF-α, IL-2) or activation markers (CD38, CD154, CD137). Representative flow analysis for a UD and CCD stimulated with combined SARS-CoV-2 peptide pools, with gating applied from a no-peptide control, is shown in **Figure 2A**. The percentage of SARS-CoV-2 VSTs in CCD positively correlated (Pearson correlation p = 0.0381, r = 0.5391) with SARS-CoV-2 antibody level (**Figure 2B**). Interestingly, the percentage of SARS-CoV-2 VSTs in CCD was found to decline significantly over time (Pearson correlation p = 0.0021, r = 0.6275) (**Figure 2C**). The mean percentage of CD3+ cells expressing IFN-γ, TNF-α and CD154 (**Figure 2D**) was significantly higher in CCD compared to UD for stimulation with each individual peptide pool and also for the combined peptide pools. CCD T cell IFN-γ, TNF-α and CD154 responses to individual peptide pools (n = 10) was compared using repeated measures (RM) one-way ANOVA to determine whether there was a preferential response to specific SARS-CoV-2 antigens. While the mean percentage of CD3+/IFN-γ+ cells and CD3+/TNF-α+ cells was comparable between the three peptide pools, the CD4+/CD154+ response was significantly higher (p = 0.042) to membrane peptides than to nucleocapsid peptides. Altogether, these data indicate there is no consistent preferential T cell cytokine response to one particular SARS-CoV-2 antigen (**Figure S3A**).

Further dissection of the cytokine response to SARS-CoV-2 peptide pools within lymphocyte subsets CD4+ T cells, CD8+ T cells and NK cells (CD56+/CD3- PBMCs) from CCD indicates the IFN-γ response is primarily by CD4+ T cells (**Figure S3B**). The mean percentage of CD4+/IFN-γ+ PBMC was significantly higher than either CD8+/IFN-γ+ or CD56+/IFN-γ+ cells for each individual peptide pool. Stimulation with combined peptide pools drove induction of higher percentages of CD4+/IFN-γ+ T cells than either CD8+/IFN-γ+ or CD56+/IFN-γ+ cells. The percentage of CD8+/IFN-γ+ cells was significantly increased over CD56+/IFN-γ+ cells. Conversely the TNF-α response to pooled peptides demonstrated significantly higher CD56+/TNF-α+ cells than CD8+/TNF-α+ cells (**Figure S3C**).

### Isolation of SARS-CoV-2 VSTs Using Peptide-Driven IFN-γ Selection and Expansion in Culture

**Figure 3:**
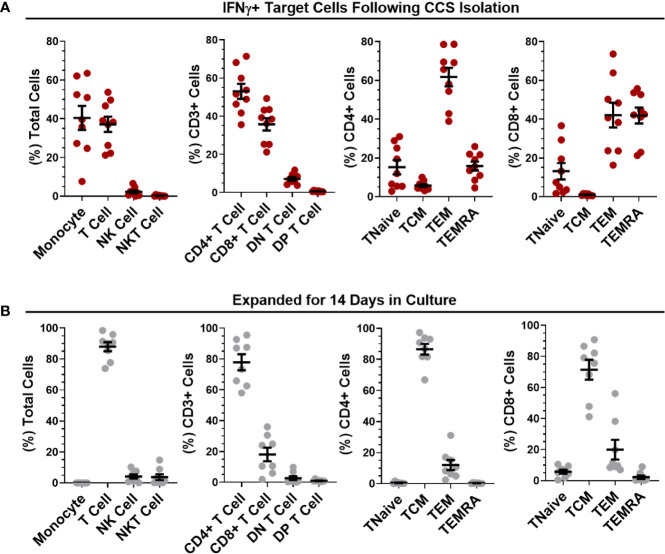
Phenotypic analysis of isolated and expanded SARS-CoV-2 VSTs. The percentages of leukocytes, T cell subpopulations and CD4/CD8 differentiation status were quantified for **(A)** IFNγ+ target cells directly after SARS-CoV-2 peptide-mediated cytokine capture system (CCS) isolation and **(B)** following expansion in culture for 14 days. All data is represented as mean ± SEM.

PBMC were stimulated with combined peptide pools and reactive VSTs were isolated with the CliniMACS IFN-γ Cytokine Capture System (CCS) kit. Analysis of the IFN-γ selected T cells (**Figure 3A**) revealed an equal ratio of monocytes to T cells with negligible levels of NK or NKT cells. CD3+ T cells in the isolated fraction were a mix of CD4+ (53.02 ± 3.94%) and CD8+ (35.73 ± 3.23%) cells; where CD4+ T cells were predominantly central memory (86.52 ± 3.44%), CD8+ T cells showed mostly effector memory and terminal effector RA (TEMRA) phenotype. The non-target cells from the CCS isolation were irradiated and co-cultured with the isolated IFN-γ+ target cells to act as feeders for VST culture expansion in G-Rex culture vessels. After 14 days expansion (**Figure 3B**), cultures were highly enriched for T cells (87.95 ± 2.99%) with minimal expansion of NK and NKT cells. T cells were predominantly CD4+ (77.86 ± 5.19%) with smaller proportion of CD8+ T cells (18.05 ± 4.4%). Both the CD4+ and CD8+ populations were heavily skewed towards central memory phenotype. Direct comparison of populations between isolation and day 14 expansion showed significant differences in monocyte and T cell content, CD4+, CD8+ and Double Negative (DN) T cells, and memory subpopulations in both the CD4 and CD8 compartment (**Figure S4A**) demonstrating an enrichment of central memory CD4 cells in our culture process. In addition, expanded VSTs showed negligible co-expression of T cell exhaustion markers PD-1 and Tim-3 in both the CD4 and CD8 compartment (**Figure S4B**) indicating the culture expansion has not induced an exhausted T cell phenotype.

### Expanded SARS-CoV-2 VSTs Show Specific Response to All SARS-CoV-2 Peptides

**Figure 4:**
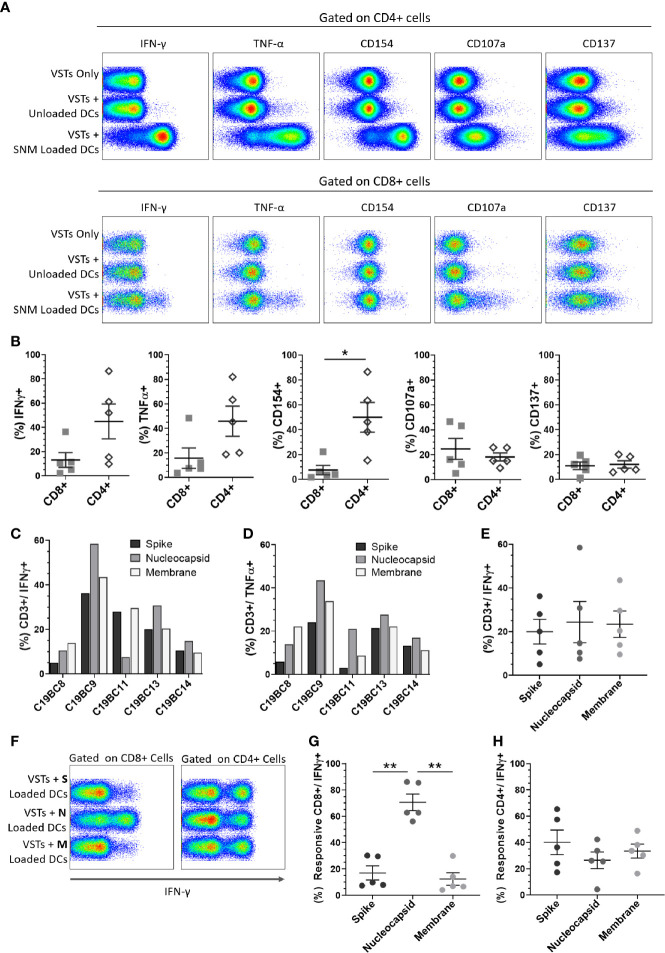
Cultured SARS-CoV-2 VST peptide specificity. Isolated and expanded SARS-CoV-2 VST (n = 5) at day 14 culture were co-cultured with peptide-loaded mature autologous DC. **(A)** Flow cytometric analysis on either CD3/CD4+ or CD3/CD8+ cells for IFN-γ, TNF-α, CD154, CD107a, and CD137 is shown for negative controls (VSTs Only, and VSTs + Unloaded DCs), and VST with pooled SARS-CoV-2 peptide loaded DCs (VST + SNM-loaded DCs). **(B)** The mean percentage of CD4+ and CD8+ positive for each marker of DC-stimulated VST was compared using paired t-test Holm-Sidak correction for multiple comparisons, *p ≤ 0.05. **(C, D)** Individual donor VST were assessed for T cell response (% CD3+/IFN-γ+ and % CD3+/TNF-α+ respectively) against DCs loaded with individual SARS-CoV-2 peptide pools: spike, nucleocapsid and membrane. **(E)** Collated responses to the individual peptides in the total CD3+ population indicated no significant difference. **(F, G)** A significantly higher CD8+/IFN-γ+ cells response was seen with nucleocapsid stimulation than with the other peptide pools (significance determined using RM one-way ANOVA with Geisser-Greenhouse correction **p ≤ 0.01). **(H)** CD4+/IFN-γ+ cells responded similarly to all the three peptide pools. All data represented as mean ± SEM.

SARS-CoV-2 peptide pool-loaded dendritic cells (DCs) and unloaded DC controls were then co-cultured with 14-day expanded VST at (1 DC: 10 VST) and analyzed for T cell activation and cytokine expression. Both CD4+ and CD8+ VSTs demonstrated specific anti-viral reactivity *via* expression of IFN-γ, TNF-α, CD154, CD107a, and CD137 when co-cultured with autologous DC loaded with SARS-CoV-2 pooled peptide (representative plots **Figure 4A**). There was a stronger response to peptide re-stimulation in CD4+ T cells than in the CD8+ T cells for IFN-γ, TNF-α, CD154 (**Figure 4B**), but equivalent CD107a and CD137 expression. The total T cell IFN-γ and TNF-α response to individual peptide pools for each donor VST are shown in **Figures 4C, D**, respectively, indicating donor-specific variation. When the data were collated, equivalent reactivity to all three peptide pools was observed (**Figure 4E**). Although the total T cell population did not show predominance for any of the SARS-CoV-2 antigen pools, the IFN-γ response was also assessed for the individual peptide pools, gated on CD8+ T cells and CD4+ T cells specifically (representative plot, **Figure 4F**). In CD4+ T cells there was a similar response to each peptide pool, but in CD8+ T cells, the nucleocapsid peptide pool was clearly immunodominant, inducing the strongest response (**Figures 4G, H**).

We assessed whether other non-SARS virus-specific T cells were coincidentally expanded in this process. Stimulation with EBV, adenovirus or irrelevant (GAD65) peptides did not demonstrate any significant levels of T cells directed to other viruses as measured by intracellular IFN-g response (**Figure S5**).

### Culture Optimization to Enhance SARS-CoV-2 VST Expansion for Clinical Manufacture

**Figure 5:**
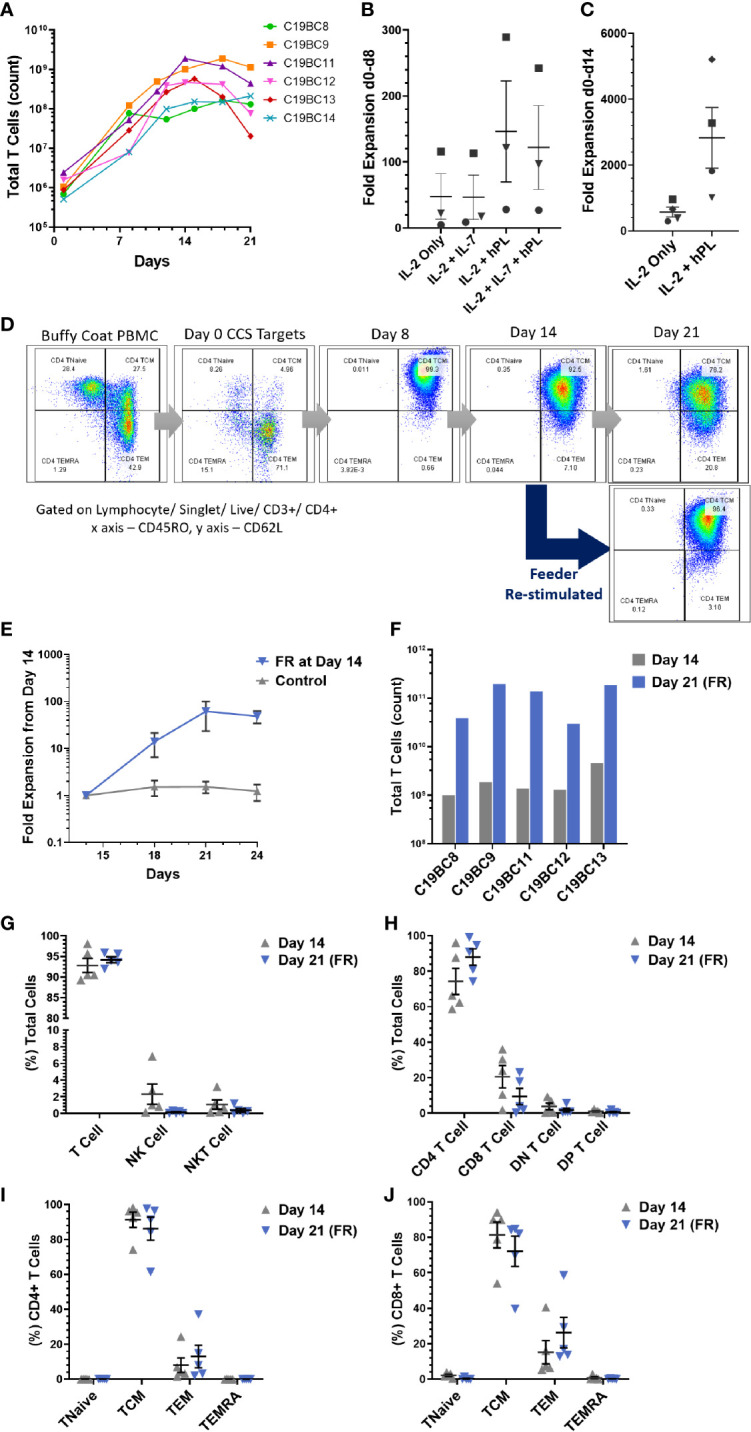
SARS-CoV-2 VST culture optimization. **(A)** Isolated SARS-CoV-2 VST from donors C19BC8-14 had a two- to three-log expansion over 21 day culture using an optimized culture expansion protocol. Variation in the start numbers of VST reflect donor variation in initial buffy coat PBMC numbers. Fold expansion between **(B)** day 0 and day 8 and **(C)** day 0 and day 14 was assessed in cultures after supplementation with IL-2, IL-7, and human platelet lysate (PL). Donors C19B9 (square), C19BC11 (triangle), C19BC12 (circle), and C19BC13 (diamond) were divided to compare medium supplementation condition. **(D)** Representative culture C19BC9 by day 21 without re-stimulation indicated some transition of CD4 TCM to CD4 TEM (Day 21 top panel). CD4 CM phenotype was retained when cultures re-stimulated at day 14 with autologous irradiated feeders (Day 21 bottom panel). **(E)** VST cultures were split at day 14 to directly compare standard continuation in culture (control) and re-stimulation with autologous irradiated feeders. Data is represented as mean T cell count ± SEM (n = 5). **(F)** VST from a single donor buffy coat were compared for optimal cell yields at day 14 (grey), and day 21 with feeder re-stimulation at day 14 (Day 21 FR, blue). **(G–J)** Final product phenotype and T cell memory status was compared at both harvest time-points: Day 14 (grey triangles) and Day 21 FR (blue triangles). Data is represented as mean ± SEM. No significant differences were observed using paired t-tests with Holm-Sidak correction for multiple comparisons.

Establishing a bank of HLA-typed, donor-derived VSTs rely upon significant cell expansion in order to provide sufficient doses for clinical trial or therapeutic treatment. Combined growth curves of VST samples C19BC8 to C19BC14 (n = 6) cultured at optimized seeding density demonstrated a two- to three-log expansion from the initial isolated IFN-γ+ cells at day 0 to day 14, followed by a general plateau in expansion beyond this point (**Figure 5A**). Additionally, some VST cultures were expanded in different medium supplements for optimization of culture conditions, supplemented with IL-2, IL-7 or commercial pathogen-inactivated human platelet lysate (hPL). Addition of IL-7 had no effect on culture expansion between day 0 and day 8 (**Figure 5B**), whereas addition of hPL induced a markedly higher fold expansion between day 0 and day 14 compared to IL-2 alone in each donor culture tested (**Figure 5C**). After day 14 culture expansion plateaued, and an increased transition from central memory to effector memory phenotype by day 21 was observed (representative plot, **Figure 5D**). When cultures were administered a second feeder cell re-stimulation (FR) with autologous irradiated cells at day 14, central memory phenotype was retained at day 21. FR induced a subsequent two log expansion between days 14 and 21 (**Figure 5E**) in all VST cultures tested (n = 5). The final VST numbers harvested under optimized conditions from a single CCD buffy coat ranged from 1 to 4.6 × 10^9^ at day 14, and 0.3 to 2 × 10^11^ at day 21 following FR (**Figure 5F**). Cultures were monitored throughout expansion to determine whether FR affected culture composition, but no significant differences in lymphocyte subsets (**Figures 5G, H**) or T cell memory status (**Figures 5I, J**) were observed between cells harvested at day 14 or at day 21.

### Scale Manufacture of Demonstrator VST Products

Successful isolation and expansion of two VST products was performed using at-scale manufacturing processes. VST from leukapheresis material was isolated using Prodigy and cultured for 14 days in closed-process flasks prior to harvest. This yielded at least 3 × 10^9^ VST and the VST demonstrated consistent phenotype and function to the developmental products (**Figure S6**).

## Discussion

In this study we have characterized the virus-specific T cell compartment in SARS-CoV-2 convalescent donors, who volunteered to donate convalescent plasma (CCD). The immunophenotyping of donor PBMC demonstrated broadly similar percentages of different immune subpopulations compared to UD, though there was a significant decrease in CD19 B cells in CCD in common with many other reports (11, 25–27). However, we identified a significant increase in the NK cell compartment in CCD. Though increased innate lymphocyte levels have been correlated with increasing age (28), there was no correlation between donor age and frequency of NK cells or B cells. There was a significant correlation between B cell level and SARS-CoV-2–specific antibody levels. The T cell compartment showed no significant differences in CD4/8 ratios or differentiation status between CCD and UD, which indicates that mild COVID-19 does not significantly affect the overall T cell composition. There is however clear evidence of double-positive T cells, associated with recent viral infections (29).

Only recovered donors with confirmed infection and mild symptoms (non-hospitalized) were investigated in this study. Samples were collected between 34 and 56 days after resolution of symptoms, and in agreement with other studies we find that in these patients a robust T cell response is generated (30) against the spike, nucleocapsid and membrane glycoprotein peptide pools. A number of reports have indicated that protein or peptides from the C terminal of the spike protein can elicit T cell responses in donors known to be SARS-CoV-2 negative, indicating a cross-reaction with conserved motifs in other coronaviruses (31, 32) but this was not observed using this spike peptide pool. There was some indication that peptide pools from different proteins elicited a differential cytokine response, with IFN-γ and TNF-α responses stronger to nucleocapsid peptides, though CD154 was preferentially increased in response to membrane peptides. This correlates well with findings in other cases of mild COVID-19 (33). The relatively low percentage of VSTs detectable has been reported in other studies on COVID-19 patients with mild disease (31). The key finding from this initial work was that we could successfully elicit IFN-γ responses in SARS-CoV-2 VSTs from CCD peripheral blood after peptide stimulation, which then confirmed that we could isolate and expand these T cells using an established clinical-grade cytokine capture assay. The principal confounding factor was identifying that SARS-CoV—2 VST levels were closely correlated with time from resolution of infection, with VSTs dropping to less than 0.03% by 60 days post-resolution of infection. Further work is ongoing to determine whether SARS-CoV-2 VST remain detectable later in convalescence, though preliminary investigations suggest residual T cell responses up to six months (34). However subsequent re-exposure to the virus may result in reinforcement and expansion of these residual cells.

Virus antigen-stimulated T cells were isolated using a GMP-compliant IFN-γ bead selection process and rapidly expanded *in vitro*. The isolated peptide-reactive T cells were predominantly differentiated effector T cells, with an equal CD4:CD8 split, but after 14-day culture, there was an overwhelming shift to central memory phenotype with a strong skew to CD4 T cells and negligible expression of T cell exhaustion markers seen in some donors (35). This correlates closely with the CD4-predominant expanded populations produced using the methods described by Keller et al. (36). This change may reflect a loss of effector T cells and a rapid expansion of the central memory (TCM) compartment. The cultured VSTs also retain strong specificity for viral peptides as co-culture with autologous peptide-loaded DC drives a pronounced CD4 activation and cytokine response, indicating that these expanded T cells retain proliferative capacity. Interestingly, the CD8 response was significantly lower than CD4 VSTs, with low expansion and weaker responses to the SARS-CoV-2 antigens. This reduced CD8 response may be an advantage for a therapeutic product, as in models there is a clear protective role for CD8 T cells against acute SARS infection (37), but there is evidence that hyper-activation of CD8 T cells can be linked to severity of COVID-19 disease (38). The expanded CD8 VST cell demonstrated differential responses to each protein peptide pool and there was clear indication that the nucleocapsid protein is the immunodominant antigen for the cytotoxic T cell population.

Adoptive anti-viral T cell therapy has been an important therapeutic approach for other infections such as EBV, CMV and adenovirus (20, 22, 39). Various manufacturing methods have been developed, but cytokine capture has proved effective for isolation of T cells for clinical therapy (17–19). Other methods for isolation and/or expansion of antigen-specific T cells have been developed, including isolation of tetramer-binding T cells (40), selection of activated T cells post antigen-exposure using CD137 selection (41), and expansion of virus-specific T cells through optimized *in vitro* stimulation with peptides and cytokines (36).

Direct “collect and select” methods such as tetramer selection or cytokine-based selection followed by infusion without expansion do have advantages in terms of rapidity of manufacture, but are very limited in the doses of T cells that they yield. Selection by surface marker such as CD137 has potential advantages in that it is not restricted to a subset of cells making one cytokine, but requires pre-depletion of interfering components such as CD25+ regulatory cells, and also selects activated NK cells, so may still require substantial manipulation and potential cell losses to achieve a memory T cell product (41).

The dose of cells required to treat SARS-CoV-2 infection is currently unknown, however treatment of patients where rising viremia is targeted such as cytomegalovirus (CMV) and adenovirus (AdV), have used total doses of up to 10^7^ cells and single or few repeat doses (19, 40). Whether interventions established for these DNA viruses will be applicable to an RNA virus remains to be determined. We are concentrating on manufacturing an HLA-typed bank of donated cells, as even a modest manufacturing process would yield 100 to 200 doses at a target dose of 10^7^ cells per treatment. This approach could also be achieved using the recently published methods (also based on previous work with other virus infections such as CMV/ADV) by Keller et al. in October 2020 (36).

A VST product based on this manufacturing method, or others currently in development (42), will require careful First in Human clinical trials to determine the best route for use of VST in SARS-CoV-2 infection. Patients treated with VSTs for CMV- or Epstein-Barr Virus (EBV)-mediated disease are commonly immune-suppressed as a result of the treatment pathway for stem cell or solid organ transplantation (19–21). A rapidly increasing CMV titer against a background of leukopenia, or proliferating EBV-infected lymphoma cells in an immune-suppressed solid organ graft patient will present differently to COVID-19 infection in terms of homeostasis for allogeneic T cell engraftment and antigen availability to stimulate T cell expansion. First clinical indications would be likely be in patients who are already immune-suppressed, capable of accepting a T cell graft, and at increased risk of severe COVID-19 disease. Recent evidence in the cancer field suggests that T cells up to 1 × 10^9^ per dose (43) can be introduced with indicators of *in vivo* efficacy with no prior leukodepletion – this may also support the introduction of SARS-CoV-2 VSTs in “at risk” individuals in early COVID-19 infection without conditioning. SARS-CoV-2–specific VST may also be supportive in patients with current COVID-19 infections who are at high risk of increased disease severity due to pre-existing co-morbidities or who have susceptible immunotypes (44), and protect against exacerbation of infection.

The initial 14-day culture of SARS-CoV-2 VSTs in GMP-compliant reagents demonstrated that a suitable therapeutic T cell product could be manufactured from even small numbers of VSTs present in a single unit of blood, yielding up to 5 × 10^9^ cells per manufacturing run with greater than 90% central memory T cells and a 3.3 log expansion. Further expansion was provided by a second round of stimulation with autologous feeder cells, resulting in an approximate 2-log further increase in cell numbers with a consolidation of central memory phenotype, plus reduction in NK cells. There was a further skew towards CD4 T cells during this second expansion. Thus, scaling up to a full manufacturing process using lymphocyte-optimized leukapheresis instead of buffy coats, with GMP-compliant isolation using CliniMACS Prodigy cell processing and expansion in closed-culture G-Rex culture flasks we could generate sufficient material to treat multiple patients from a single suitably HLA-matched donor, with or without a second expansion phase. In this study donors were not selected based on HLA type. However, HLA typing of consented donors revealed the presence of HLA class I and II alleles common in the UK population and donor genotypes contained alleles known to present multiple SARS-CoV-2 peptides *in silico* (23, 45). As further information becomes available on HLA allele/peptide binding, donors could be selected for optimal VST efficacy on the basis of HLA type which would clarify which loci and alleles should be matched between a T cell donor and patient for best effect. HLA matching is important for effective function of adoptive T cell therapies, and it is clear that some HLA types are poorer at presenting SARS-CoV-2 peptides such as B*46:01 (45). However, the donor HLA range seen in **Table 1** indicates HLA subtypes with strong peptide-presenting capacity such as A*02:01 and DRB*15:01, so supplementation of immune response with donor T cells from a matched donor could be a therapeutic option where few others exist. Therefore, we demonstrate the feasibility of generating large quantities of virus-specific T cell products for clinical trials in support of severe SARS-CoV-2 infections where the endogenous T cell response is compromised, representing a potentially significant advance in therapy for COVID-19 (46).

## Limitations of This Study

This study has used the three SARS-CoV-2 antigen peptide pools (Spike, Nucleocapsid and Membrane) available from April 2020 to generate the phenotyping and expansion data of antigen-specific T cells. Although quality tested, the peptide pools used in this study are research grade and require reconstitution and filtration. GMP-grade peptide pools designed for unmanipulated use with e.g. the prodigy system should be adopted once available. Additional antigens may reveal a fuller picture of the immune response in COVID-19, and their addition to the T cell expansion method described here could have a positive additive effect. However this could also increase the degree of cross-reaction with other non-SARS coronavirus-specific T cells. The demographics of the local donor pool used is necessarily limited to blood donors who have undergone mild COVID-19 disease. Different T cell responses and proliferation characteristics may be found in other donor populations recovering from a range of COVID-19 symptoms. Clinical use of a VST product based on this manufacturing strategy will require controlled clinical trials for safety and efficacy assessment.

## Data Availability Statement

The original contributions presented in the study are included in the article/**Supplementary Material**. Further inquiries can be directed to the corresponding author.

## Ethics Statement

The studies involving human participants were reviewed and approved by SNBTS Sample Governance Committee, SNBTS. The patients/participants provided their written informed consent to participate in this study.

## Author Contributions

RC, AF, MT, and JC conceived and designed the study. RC, AF, MT, and JC drafted and revised the manuscript. RC, AF, LS, PB, SI, and DT performed experimental work. RC, AF, LS, PB, SI, DT, and JC analyzed data. LJ, SZ, and MT performed clinical tasks including identification, consenting, and testing of donors. All authors contributed to the article and approved the submitted version.

## Funding

This work was funded by NHS National Services Scotland. The work was funded in part by a Medical Research Scotland COVID-19 research grant CVG-1716-2020, awarded to RC and AF.

## Conflict of Interest

The authors declare that the research was conducted in the absence of any commercial or financial relationships that could be construed as a potential conflict of interest.
