# Supplementary material for: Rapid GMP-Compliant Expansion of SARS-CoV-2–Specific T Cells From Convalescent Donors for Use as an Allogeneic Cell Therapy for COVID-19
Source: Front Immunol. 2021 Jan 8;11:598402. doi: 10.3389/fimmu.2020.598402 (PMC7819874; doi:10.3389/fimmu.2020.598402)
Supplement: Supplementary file 1 [file DataSheet_1.docx]

**Supplementary Figures**

|  | Amino Acid Sequence |
| --- | --- |
| Peptivator SARS-CoV-2 Spike (Protein QHD43416.1) | MFVFLVLLPLVSSQCVNLTTRTQLPPAYTNSFTRGVYYPDKVFRSSVLHSTQDLFLPFFSNVTWFHAIHVSGTNGTKRFDNPVLPFNDGVYFASTEKSNIIRGWIFGTTLDSKTQSLLIVNNATNVVIKVCEFQFCNDPFLGVYYHKNNKSWMESEFRVYSSANNCTFEYVSQPFLMDLEGKQGNFKNLREFVFKNIDGYFKIYSKHTPINLVRDLPQGFSALEPLVDLPIGINITRFQTLLALHRSYLTPGDSSSGWTAGAAAYYVGYLQPRTFLLKYNENGTITDAVDCALDPLSETKCTLKSFTVEKGIYQTSNFRVQPTESIVRFPNITNLCPFGEVFNATRFASVYAWNRKRISNCVADYSVLYNSASFSTFKCYGVSPTKLNDLCFTNVYADSFVIRGDEVRQIAPGQTGKIADYNYKLPDDFTGCVIAWNSNNLDSKVGGNYNYLYRLFRKSNLKPFERDISTEIYQAGSTPCNGVEGFNCYFPLQSYGFQPTNGVGYQPYRVVVLSFELLHAPATVCGPKKSTNLVKNKCVNFNFNGLTGTGVLTESNKKFLPFQQFGRDIADTTDAVRDPQTLEILDITPCSFGGVSVITPGTNTSNQVAVLYQDVNCTEVPVAIHADQLTPTWRVYSTGSNVFQTRAGCLIGAEHVNNSYECDIPIGAGICASYQTQTNSPRRARSVASQSIIAYTMSLGAENSVAYSNNSIAIPTNFTISVTTEILPVSMTKTSVDCTMYICGDSTECSNLLLQYGSFCTQLNRALTGIAVEQDKNTQEVFAQVKQIYKTPPIKDFGGFNFSQILPDPSKPSKRSFIEDLLFNKVTLADAGFIKQYGDCLGDIAARDLICAQKFNGLTVLPPLLTDEMIAQYTSALLAGTITSGWTFGAGAALQIPFAMQMAYRFNGIGVTQNVLYENQKLIANQFNSAIGKIQDSLSSTASALGKLQDVVNQNAQALNTLVKQLSSNFGAISSVLNDILSRLDKVEAEVQIDRLITGRLQSLQTYVTQQLIRAAEIRASANLAATKMSECVLGQSKRVDFCGKGYHLMSFPQSAPHGVVFLHVTYVPAQEKNFTTAPAICHDGKAHFPREGVFVSNGTHWFVTQRNFYEPQIITTDNTFVSGNCDVVIGIVNNTVYDPLQPELDSFKEELDKYFKNHTSPDVDLGDISGINASVVNIQKEIDRLNEVAKNLNESLIDLQELGKYEQYIKWPWYIWLGFIAGLIAIVMVTIMLCCMTSCCSCLKGCCSCGSCCKFDEDDSEPVLKGVKLHYT |
| Peptivator SARS-CoV-2 Nucleocapsid (Protein QHD43419.1) | MSDNGPQNQRNAPRITFGGPSDSTGSNQNGERSGARSKQRRPQGLPNNTASWFTALTQHGKEDLKFPRGQGVPINTNSSPDDQIGYYRRATRRIRGGDGKMKDLSPRWYFYYLGTGPEAGLPYGANKDGIIWVATEGALNTPKDHIGTRNPANNAAIVLQLPQGTTLPKGFYAEGSRGGSQASSRSSSRSRNSSRNSTPGSSRGTSPARMAGNGGDAALALLLLDRLNQLESKMSGKGQQQQGQTVTKKSAAEASKKPRQKRTATKAYNVTQAFGRRGPEQTQGNFGDQELIRQGTDYKHWPQIAQFAPSASAFFGMSRIGMEVTPSGTWLTYTGAIKLDDKDPNFKDQVILLNKHIDAYKTFPPTEPKKDKKKKADETQALPQRQKKQQTVTLLPAADLDDFSKQLQQSMSSADSTQA |
| Peptivator SARS-CoV-2 Membrane (Protein QHD43423.2) | MADSNGTITVEELKKLLEQWNLVIGFLFLTWICLLQFAYANRNRFLYIIKLIFLWLLWPVTLACFVLAAVYRINWITGGIAIAMACLVGLMWLSYFIASFRLFARTRSMWSFNPETNILLNVPLHGTILTRPLLESELVIGAVILRGHLRIAGHHLGRCDIKDLPKEITVATSRTLSYYKLGASQRVAGDSGFAAYSRYRIGNYKLNTDHSSSSDNIALLVQ |

**Supplementary Table 1.** SARS-CoV2 15mer peptide pools overlapping by 11 amino acids corresponding to spike, membrane and nucleocapsid regions were derived from Genebank sequence MN908947.3 and purchased from Miltenyi Biotec. Preservative-free peptide pools, >70% purity, QA batch-released by Miltenyi Biotec, with defined endotoxin levels. This translated to an Endotoxin burden of <0.18 EU/ml when used to stimulate T cells in the peptide batches used in this study. We first reconstituted these products in sterile water/DMSO then the peptide pools were sterile filtered using a 0.22μm filter. The peptides were sterile-filtered again immediately before addition to the PBMC cultures. Peptides were used in assays at a final concentration of [0.3 nmol/ mL].


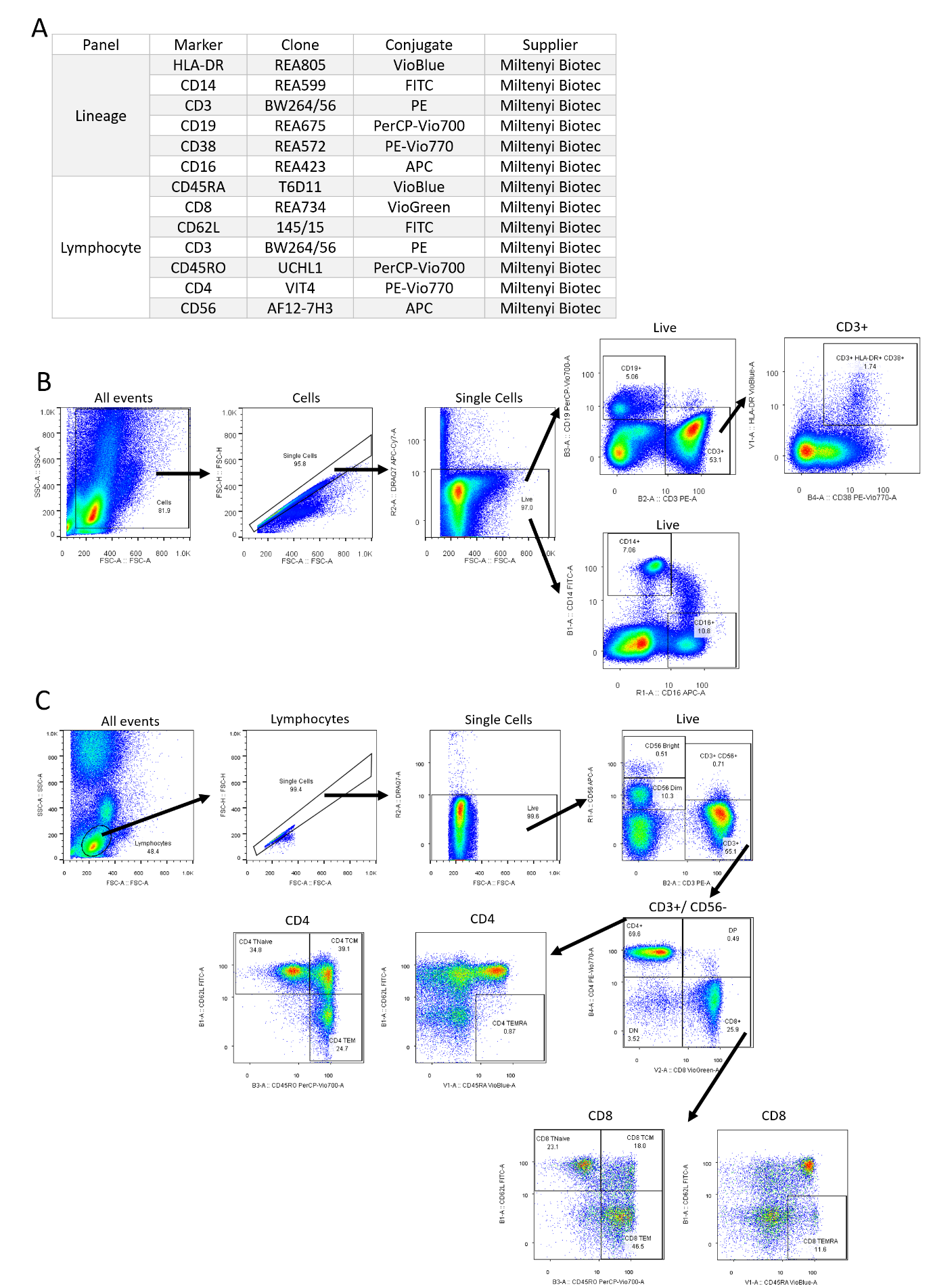


**Figure S1.** Surface phenotyping panels and gating strategies. **(A)** Antibodies used in surface marker panels. **(B)** Gating strategy for Lineage panel analysis. **(C)** Gating strategy for Lymphocyte panel analysis.


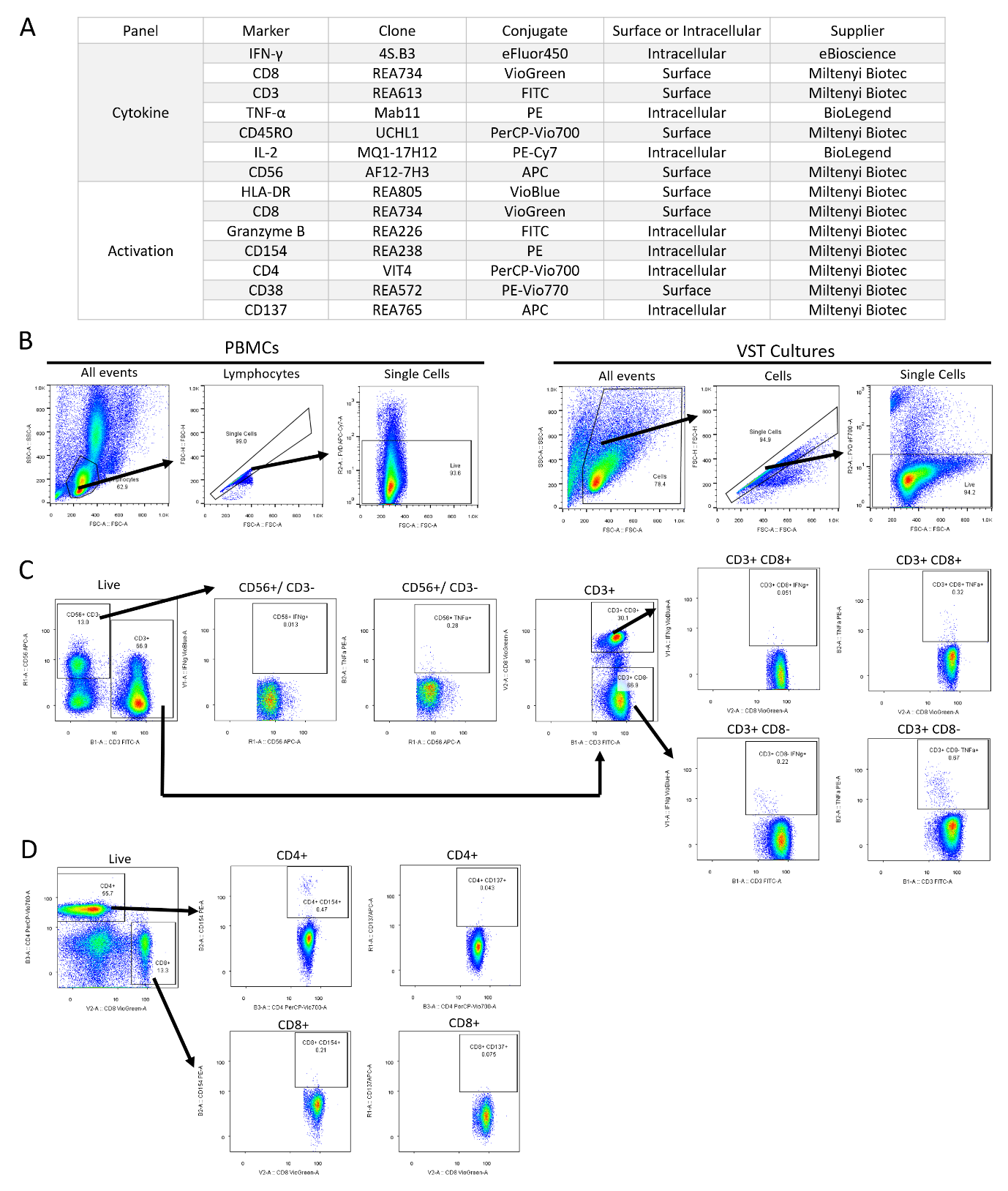


**Figure S2.** Intracellular flow cytometry panels and gating strategies. **(A)** Antibodies used in cytokine and activation panels. **(B)** All analyses were subject to initial sequential gating as shown for PBMCs and VST cultures. Flow cytometry gating strategies following initial gating above for **(C)** cytokine and **(D)** activation panel are shown using representative PBMC.

**Figure S2.** Intracellular flow cytometry panels and gating strategies.


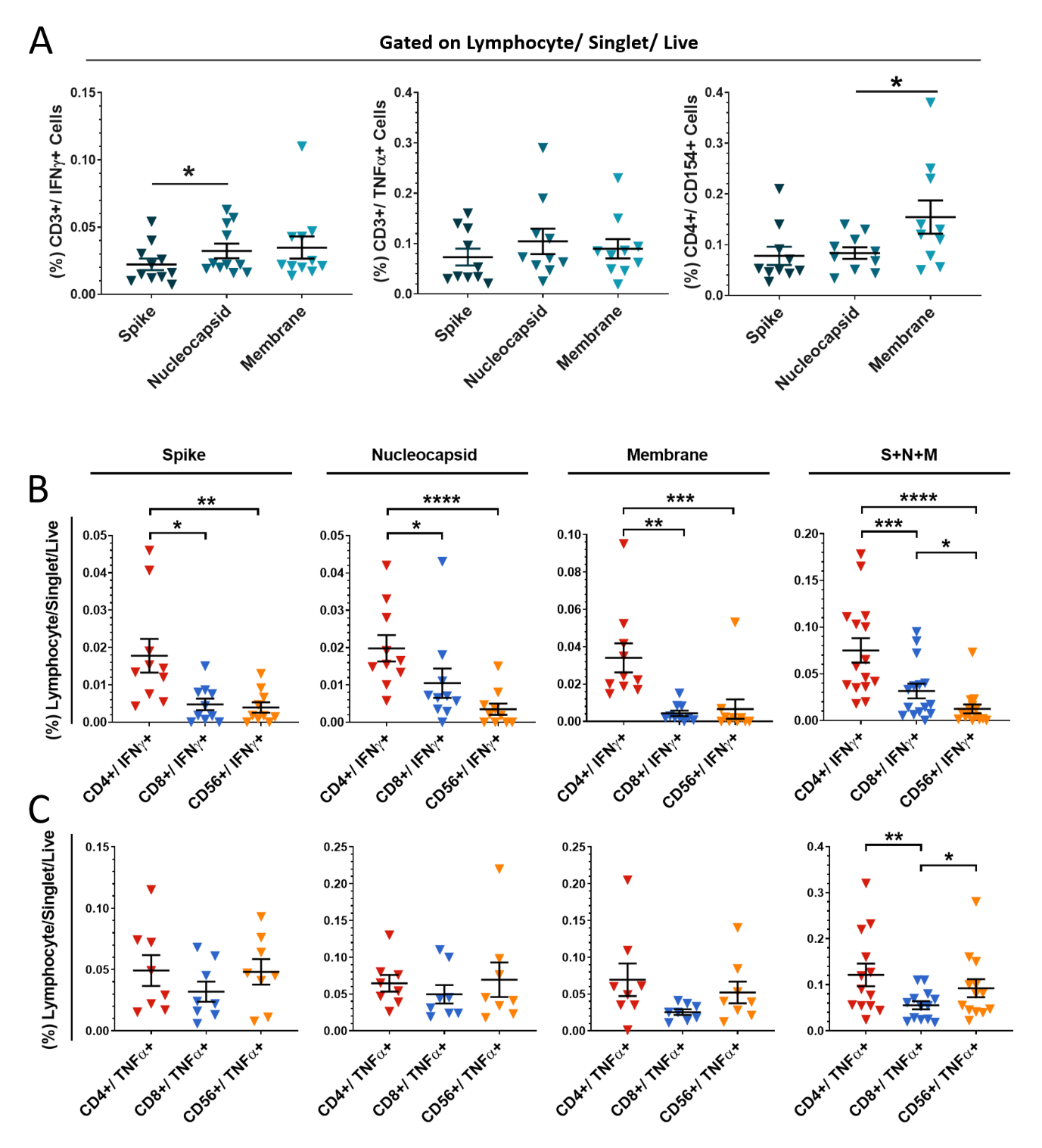


**Figure S3.** COVID-19 convalescent donor PBMC responses to SARS-CoV-2 peptides. **(A)** Mean percentages of CD3+/IFN-γ+ cells, CD3+/TNF-α+ cells and CD4+/CD154+ cells were compared for response to individual peptide pools: Spike, Nucleocapsid and Membrane in CCD (n=10). Data is represented as mean ± SEM. Lymphocyte subsets (CD4+ T cell, CD8+ T cell and CD56+ NK cells) were compared for **(B)** IFN-γ response and **(C)** TNF-α response stimulation with individual peptide pools and combined SARS-CoV-2 peptide pools. Data is represented as mean ± SEM. All significance was determined using RM one-way ANOVA with Geisser-Greenhouse correction where *p≤0.05, **p≤0.01, p≤0.001 and **** p≤ 0.0001.

**
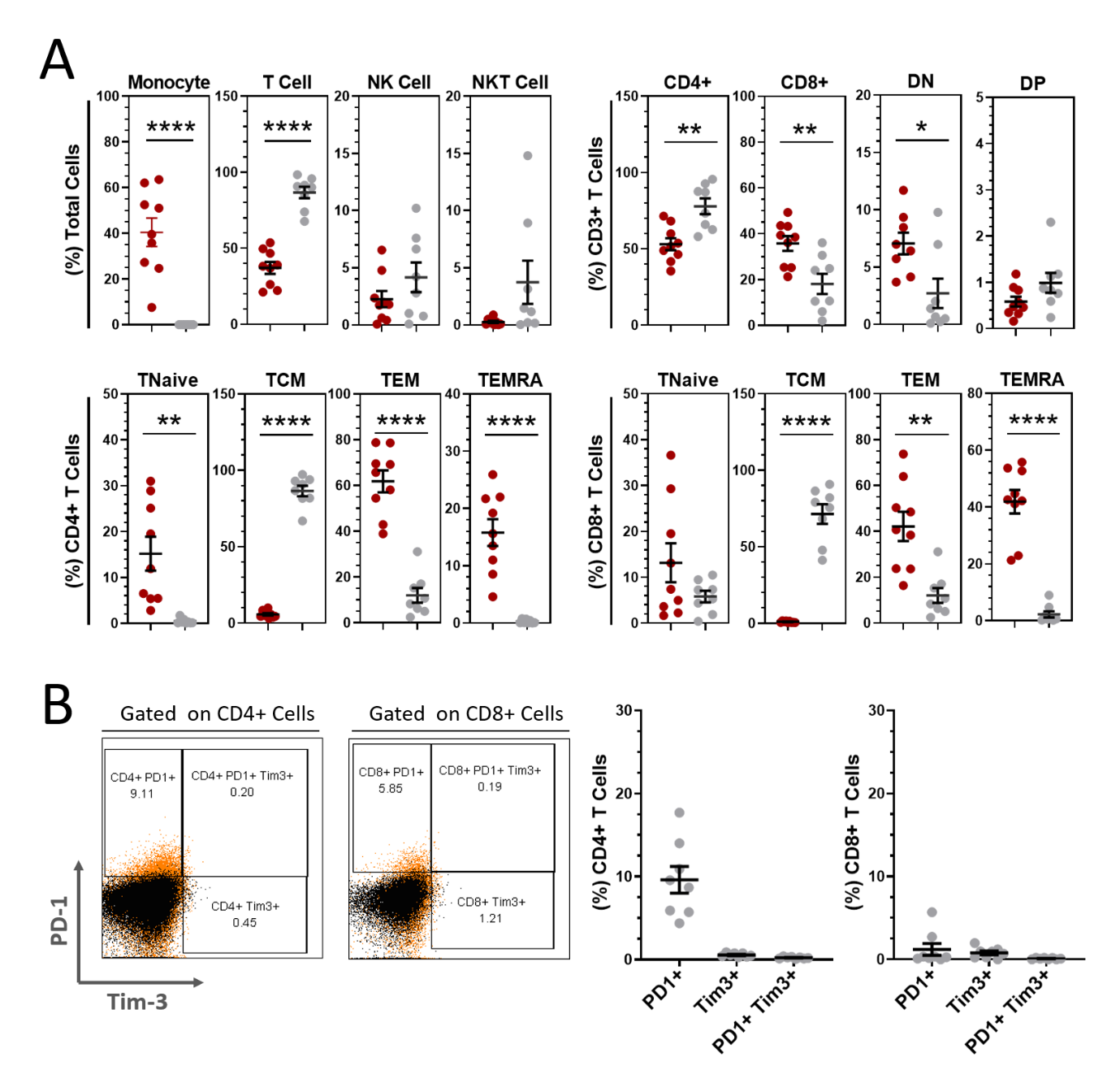
Figure S4**. SARS-CoV-2 VST phenotyping. **(A)** Direct comparison of T cell subpopulations between day 0 isolated VST and day 14 expanded VST. Significance was calculated using paired t-tests with Holm-Sidak correction for multiple comparisons *p≤0.05, **p≤0.01, p≤0.001 and **** p≤ 0.0001. **(B)** Day 14 expanded VST were also characterised for single and dual expression of exhaustion markers PD-1 and Tim-3 (orange) against isotype controls (black). All data is represented as mean ± SEM.


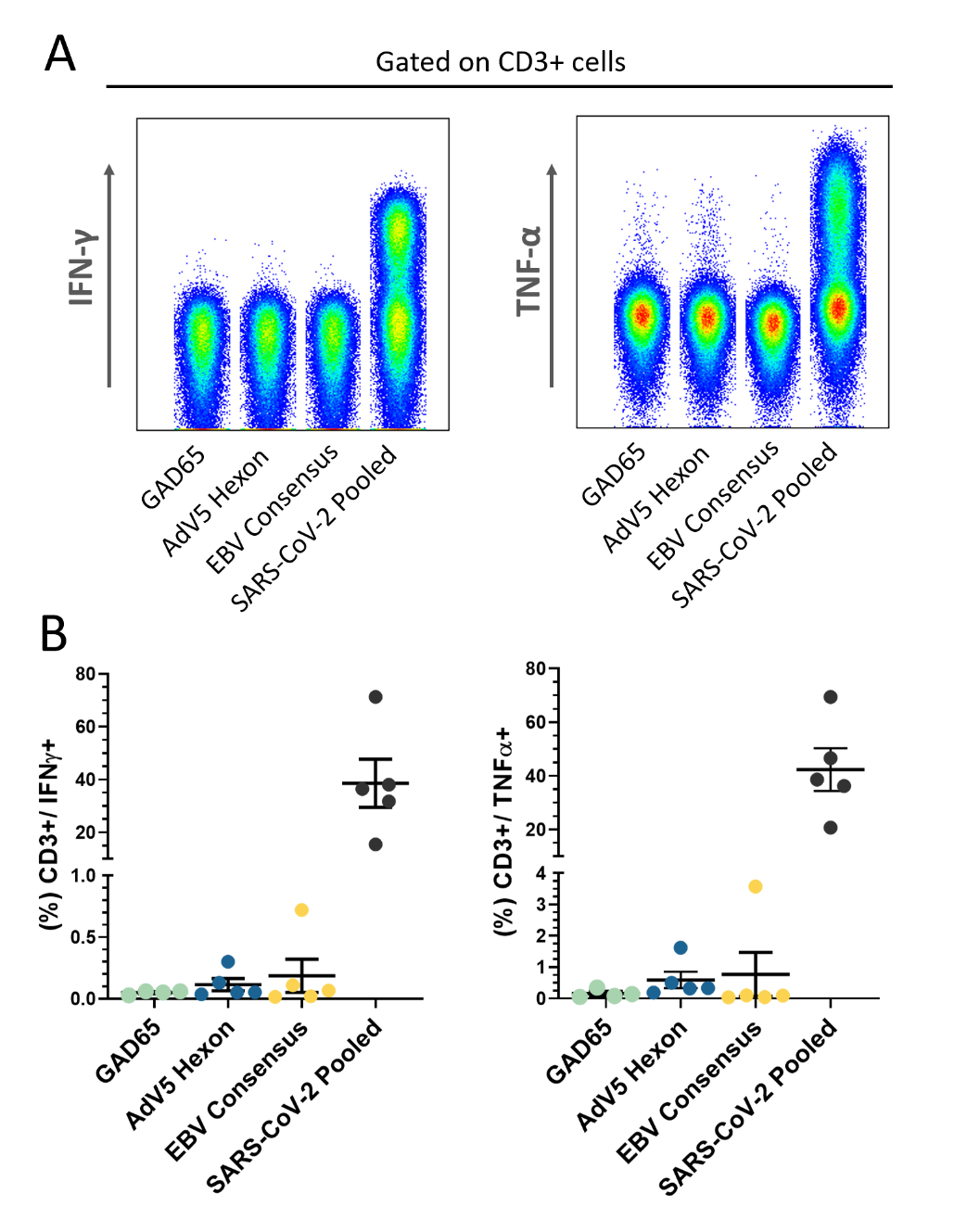


**Figure S5.** Specificity of cultured SARS-CoV-2 VSTs. SARS-CoV-2. VST cultures at day 14 were co-cultured with antigen-loaded mature autologous DCs and assessed for response. **(A)** Peptide specificity in a representative individual VST culture was assessed using DC loaded with GAD65 peptide, Adenovirus5 (AdV5) Hexon peptide, Epstein-Barr Virus (EBV) consensus peptide and combined pools of SARS-CoV-2 peptides (Spike + Nucleocapsid + Membrane) as positive control. **(B)** The mean percentage ± SEM of CD3+/IFN-γ+ cells, CD3+/TNF-α+ cells in day 14 SARS-CoV-2 VSTs (n=5) for each peptide. No statistical significance was determined.


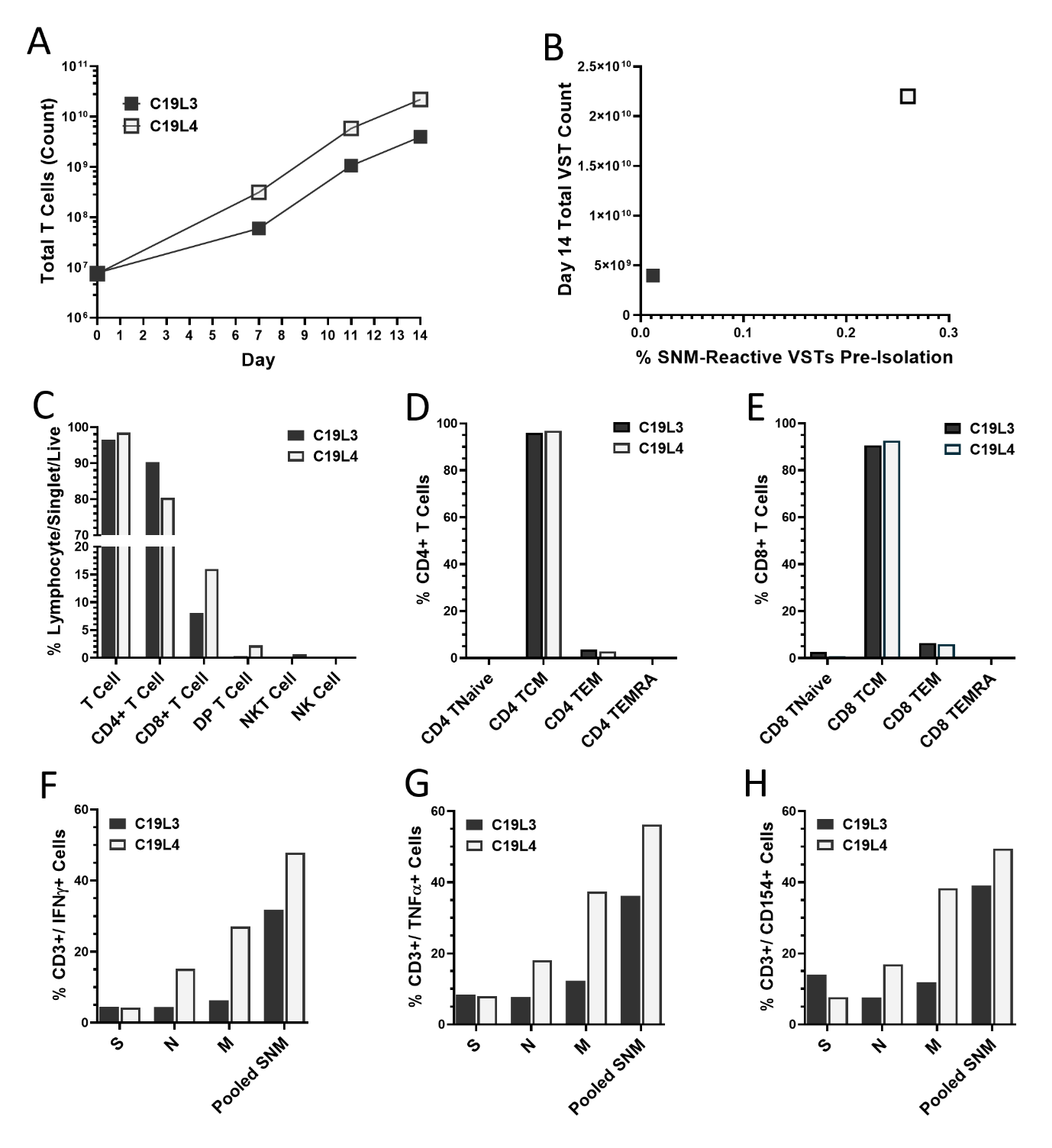


**Figure S6.** Demonstrator full scale manufacture products. Full scale VST products were manufactured using USA-derived leukapheresis donor material (n=2). SARS-CoV-2 VSTs were isolated directly from leukapheresis using CliniMACS Prodigy CCS and cultured for 14 days. **(A)** Prodigy isolated SARS-CoV-2 VST from donors C19L3 and C19L4 had a ~3 log expansion over a 14 day culture period using the optimized culture expansion protocol. **(B)** Initial flow cytometric screening on leukapheresis material identifying the frequency SARS-CoV-2 reactive VSTs (CD3+/ IFN-γ+ cells in response to pooled SNM peptide stimulation) may provide a prediction of final product yield. Final product (day 14) phenotype is shown for **(C)** lymphocyte subsets, **(D)** CD4+ T cell memory, and **(E)** CD8+ T cell memory. Final product (day 14) VST were also assessed for T cell **(F)** IFN-γ, **(G)** TNF-α, and **(H)** CD154 response against autologous DCs loaded with individual and pooled SARS-CoV-2 peptides.
